# Mixed Monolayer Characteristics of Tangeretin with Stearic Acid and Dipalmitoyl Phosphatidyl Choline at the Air/Water Interface

**DOI:** 10.3390/molecules31111952

**Published:** 2026-06-04

**Authors:** Yawen Zhou, Wanqing Wang, Tianqi Jia, Yifan Zhang, Nan Wang, Baocai Xu

**Affiliations:** School of Light Industry Science and Engineering, Beijing Technology and Business University, No. 33 Fucheng Road, Beijing 100048, China

**Keywords:** tangeretin, stearic acid, dipalmitoyl phosphatidyl choline, Langmuir monolayers

## Abstract

As a natural compound with various biological activities, tangeretin (TAN) has numerous potential advantages in food and medical fields. However, its poor aqueous solubility and bioavailability limit its effective application. In this paper, the mixed monolayer characteristics of different mass fractions of TAN with stearic acid (SA) and dipalmitoyl phosphatidyl choline (DPPC) were investigated. The optimal film-forming composition was obtained by analyzing the molecular area, monolayer compressibility, shrinkage and expansion, and other thermodynamic parameters. The results indicated that there was mutual repulsion between SA and TAN molecules, and the stability of the monolayer was poor. However, TAN/DPPC mixed monolayers exhibited favorable interactions and mutual attraction between the two components. When ω_TAN_ was 0.2, the minimum excess surface pressure of the TAN/DPPC system was −17.16 mN/m, and the minimum excess Helmholtz free energy was −3413.62 J/mol. At this composition, the intermolecular attraction between DPPC and TAN was strongest, and the mixed monolayer displayed strong deformation resistance and high stability.

## 1. Introduction

**Figure 1:**
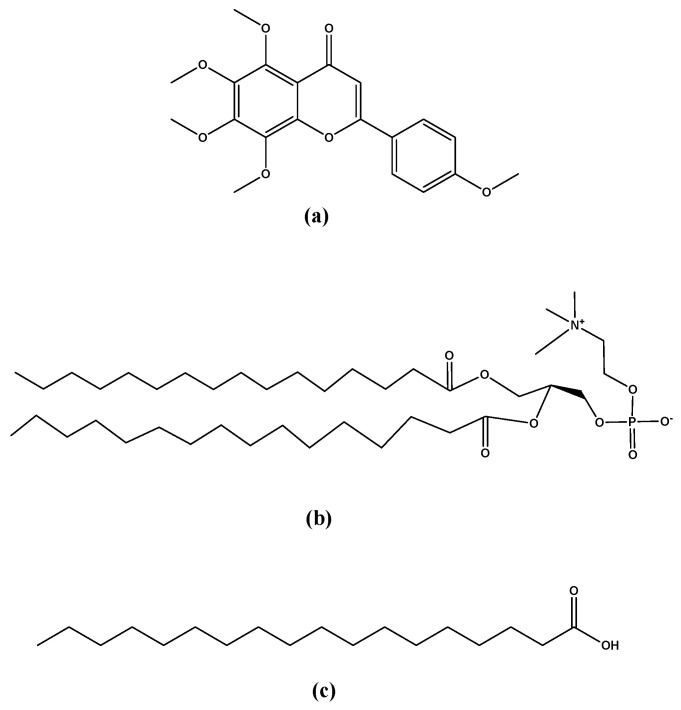
Structural formulas of TAN (**a**), DPPC (**b**) and SA (**c**).

Tangeretin (4′,5,6,7,8-pentamethoxyflavone) is a key secondary metabolite in citrus plants. It is one of the most abundant polymethoxyflavones in citrus peel. It has a flavonoid nucleus (two benzene rings connected by a pyran ring) and five methoxy substituents, and the structure is shown in Figure 1. This structure confers significant monolayer permeability and antioxidant capacity [1,2], attracting considerable attention in food and medical fields. In the food industry, the antioxidant, antibacterial, and lipid-regulation properties of tangeretin have important applications in the construction of food preservation systems and the development of functional foods [3,4,5]. In the field of medicine, tangeretin shows multi-target regulation characteristics, including core pharmacological activities such as anti-inflammatory, antioxidant and anti-tumor effects [6,7,8,9,10]. Especially in the treatment of metabolic diseases, it alleviates diabetic complications by regulating blood glucose and lipid metabolism [11]. Therefore, tangeretin has promising application prospects in the fields of food and medicine owing to its unique structural properties and diverse pharmacological effects [12].

However, its high crystallinity and poor aqueous solubility result in low oral bioavailability and limited compatibility with water-based food systems, significantly restricting its applications in medicine and functional foods [13]. At present, its bioavailability can be improved by optimizing the delivery system. The main approaches include an emulsion delivery system [14], lipid encapsulation [15], polysaccharide encapsulation delivery [16], and microencapsulation [17]. However, the accuracy and long-term safety of metabolic regulation still need to be addressed. For example, although nanostructured lipid carriers can improve bioavailability, they face the problems of complex carrier preparation and unclear metabolic pathways in vivo. The emulsion delivery strategy, e.g., hydroxypropyl methylcellulose coating, achieves >90% retention of in vitro activity, but its in vivo targeting efficiency and clinical translation potential still need to be further evaluated [18]. Therefore, the construction of an innovative strategy with efficient delivery, metabolic stability and production feasibility has become the core research direction to break through the bottleneck of clinical translation of tangeretin.

Langmuir monolayer technology has unique advantages in constructing mixed monolayer systems and simulating cell monolayer studies [19,20], which can be used to improve the stability of systems with poor biocompatibility. For example, the interaction between sakuranetin and unsaturated phospholipids showed that 1-palmitoyl-2-oleoyl-sn-glycerol-3-phosphate ethanolamine (POPE) was effectively incorporated into sakuranetin, which provided an effective insight into the development and use of sakuranetin against pathogenic factors [21]. Mixed films of curcumin with dipalmitoyl phosphatidyl choline and cholesterol were studied, clarifying the compatibility of curcumin with cell monolayer components and the optimal conditions for their preparation [22,23]. Phospholipid complexes of various substances such as a-tocopherol acetate [24] and thymol [25] have been developed. Dipalmitoyl phosphatidyl choline (DPPC) and stearic acid (SA) are good film-forming materials. As shown in Figure 1, the DPPC molecule has an amphiphilic structure, and its electroneutral characteristics and chemical inertness make it a core material for liposome construction [26,27]. In the aqueous phase environment, its acyl chain spontaneously assembles into a lamellar liquid crystal structure through hydrophobic interaction [28]. SA is a natural saturated fatty acid, and its low surface energy gives it unique interface self-assembly characteristics. The structure is shown in Figure 1. SA-modified nano-delivery systems have high stability, controllable drug release, targeted drug delivery and high customization and have been widely used in the field of drug delivery [29,30,31]. The distinct structural characteristics of SA (single hydrophobic chain, small headgroup) and DPPC (two hydrophobic chains, large headgroup) make them ideal model lipids for investigating how TAN interacts with different carrier matrices. A comparative study of these two systems can therefore provide guidance for the rational selection of lipid components in tangeretin delivery systems. Successful construction of mixed monolayers of tangeretin and other substances for application in drug delivery systems could provide new strategies for the treatment of diseases such as cancer. After the formation of mixed films, the activity of tangeretin can be better protected, so that it can play a stable role in the organism, and the tangeretin can be continuously transported to diseased cells to maintain the drug concentration within the effective range, so as to avoid side effects caused by high drug concentration and poor efficacy caused by low concentration. However, research on their application in tangeretin delivery systems remains absent.

In this study, we employed Langmuir monolayer technology to investigate the interfacial behavior of TAN/SA and TAN/DPPC mixed monolayers at various mass fractions. Surface pressure–area (π-A) isotherms and compression modulus–surface pressure (Cs^−1^-π) curves were recorded to characterize the mixed films. From these data, we determined the molecular packing of tangeretin at the interface, the thermodynamic properties of its mixed films with SA or DPPC, and the nature of the intermolecular interactions between TAN and the two lipid components. These findings establish the optimal conditions for fabricating TAN-containing mixed monolayer systems and provide a theoretical foundation for the efficient utilization of tangeretin.

## 2. Results and Discussion

### 2.1. π-A Isotherms

**Figure 2:**
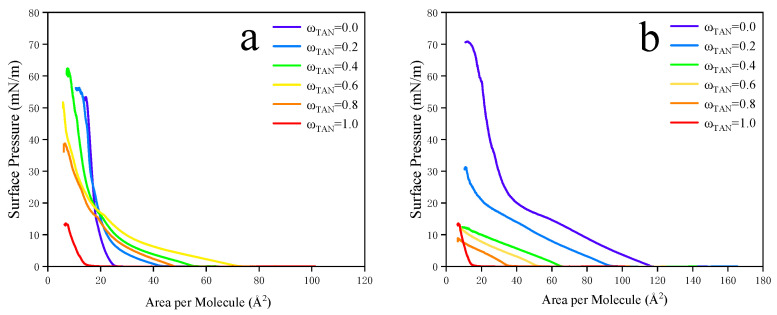
π-A isotherms of mixed monolayers: (**a**) TAN/SA, (**b**) TAN/DPPC.

Figure 2a,b are the π-A isotherms of TAN/SA and TAN/DPPC mixed monolayers, respectively. ω_TAN_ = 0.0 and ω_TAN_ = 1.0 are the π-A isotherms of pure stearic acid or pure dipalmitoyl phosphatidyl choline and pure TAN. The other curves are the π-A isotherms of TAN/SA or TAN/DPPC mixed systems with different TAN mass fractions.

From Figure 2a, it can be seen that, at lower surface pressure, with an increase in TAN mass fraction, the π-A isotherms of the mixed film first shifted slightly to the right, and the molecular arrangement in the mixed film became looser, which is very similar to the swelling behavior observed in chitosan–stearic acid mixed monolayers by Ikbal et al. [19]. At higher surface pressure, with an increase in TAN mass fraction, the isotherm shifted slightly to the left and gradually approached the isotherm of pure TAN. The molecular arrangement in the mixed film became tight, and the membrane cohesion increased. A distinct plateau emerged at ~15 mN/m when 0.4 ≤ ω_TAN_ ≤ 0.8, suggesting the coexistence of liquid-expanded and liquid-condensed phases, with TAN likely promoting phase separation in this system.

From Figure 2b, with increasing TAN mass fraction, all isotherms shifted to the left and gradually approached the isotherm of pure TAN. This indicated that, with the addition of TAN, the molecules in the mixed film were more closely arranged and the film cohesion was increased. Xu et al. [32] obtained similar results in the study of a curcumin and DPPC mixed system. The curve for ω_TAN_ = 0.0 is the π-A isotherm of pure DPPC. As the surface pressure increased, the curve began to rise slowly, and a very obvious platform area appeared between 14 and 18 mN/m, which is also a typical feature of the DPPC isotherm [33]. At this time, the DPPC monolayer was in the transition stage from the liquid-expanded film to the liquid-condensed film. Upon addition of TAN (ω_TAN_ = 0.2), this plateau disappeared, and the curve shape changed markedly, indicating a global condensing effect without obvious phase separation.

The two mixed systems are thus affected differently by TAN, which may be related to the inherent structures of SA and DPPC. DPPC has two hydrophobic chains and a large head group. There is a larger steric hindrance between the molecules, and it has a larger single-molecule area. TAN has strong hydrophobicity, and there are p-π conjugated bonds between TAN and DPPC, so TAN can easily insert into the hydrophobic chain region of DPPC. However, SA has only one hydrophobic chain, and the head group is small. It is difficult for TAN to insert into the hydrophobic region of SA. A deeper quantitative analysis of this difference is provided later (Section 2.4 and Section 2.5).

### 2.2. Characteristic Parameters

**Figure 3:**
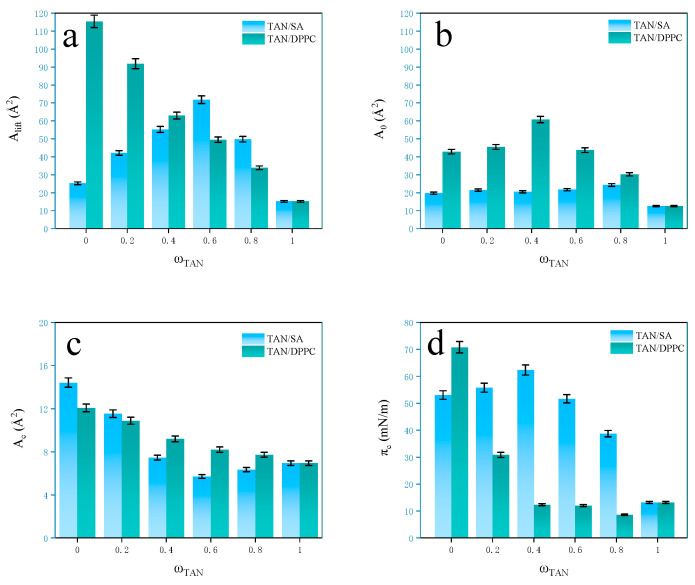
Characteristic parameters of mixed TAN/SA and TAN/DPPC monolayers: (**a**) lift-off area, (**b**) zero-pressure molecular area, (**c**) collapse area, (**d**) collapse pressure.

In order to better analyze the arrangement and compactness of mixed monolayer molecules, characteristic parameters such as areas and collapse pressure (π_c_) can be obtained from the π-A isotherm. The area parameters include lift-off area (A_lift_), zero-pressure molecular area (A_0_) and collapse area (A_c_) [34]. Figure 3 shows the characteristic parameters of TAN/SA and TAN/DPPC mixed films.

It can be seen from Figure 3a that the lift-off area of pure SA was 25 Å^2^, and the lift-off area of pure DPPC was 115 Å^2^. There is a significant difference between the lift-off area of pure SA and DPPC. The lift-off area reflects the space required for the molecules to spread freely on the subphase to form a film. The larger the lift-off area, the larger the space required for the molecules to spread freely. The reason why DPPC has a large lift-off area is that it has a molecular structure with a double long-chain alkyl hydrophobic group and a large hydrophilic group, so that the free spreading of its molecules occupies a larger space. With an increase in TAN mass fraction, the lift-off area of TAN/SA mixed films showed a trend of first increasing and then decreasing, reaching the maximum at ω_TAN_ = 0.6. For TAN/DPPC mixed films, the lift-off area gradually decreased with an increase in TAN mass fraction. Gayathri et al. [35] studied a bromofullerene–stearic acid system and also obtained a similar trend in lift-off area as observed for the TAN/DPPC mixed films.

From Figure 3b, it can be seen that the zero-pressure molecular area of pure SA was 19.8 Å^2^, and the zero-pressure molecular area of pure DPPC was 42.9 Å^2^. The zero-pressure molecular area is the area when the molecule is compressed to the maximum density. The zero-pressure molecular area of DPPC was almost twice that of SA, which indicates a smaller intermolecular distance between SA molecules and a larger area per DPPC molecule. With the addition of TAN, the content of TAN in the TAN/SA mixed films had little effect on the zero-pressure molecular area of the mixed monolayers, while the zero-pressure molecular area of the TAN/DPPC mixed films increased first and then decreased with an increase in TAN mass fraction. When ω_TAN_ = 0.4, the zero-pressure molecular area of the TAN/DPPC mixed films reached a maximum of 61 Å^2^, indicating that, when the TAN content was 40%, the molecules in the mixed film were relatively loose, and the distance between molecules was the largest. Combined with Figure 3a,b, it can be seen that the lift-off area of pure TAN was 15.2 Å^2^, and the zero-pressure molecular area was 12.6 Å^2^. The change in the zero-pressure molecular area was smaller than that in lift-off area. However, in the TAN/DPPC system the zero-pressure molecular area changed considerably, further indicating strong interactions between the two components.

Combining Figure 3c,d, it can be seen that, in the TAN/SA mixed system, as the TAN content increased, the collapse area of the mixed films first decreased and then slightly increased, while the collapse pressure first increased and then decreased. The collapse area of the mixed system in the range of ω_TAN_ = 0.4–0.6 reached the minimum value of about 7 Å^2^, and the collapse pressure was relatively higher near 57 mN/m. At this time, the mixed films had higher stability and better strength. Notably, for ω_TAN_ > 0.6, the slight increase in collapse area together with the decrease in collapse pressure may reflect partial desorption of film-forming material from the air–water interface, suggesting a reduction in film stability, which agrees well with the phase separation tendency discussed in Section 3.1. For TAN/DPPC mixed films, with an increase in TAN mass fraction, both the collapse area and collapse pressure of the mixed films gradually decreased. Raghavendra et al. [36] obtained similar results in their study of the mixed system of γ-oryzanol and DPPC. When ω_TAN_ = 0.2, the collapse pressure and collapse area were both large, indicating that the mixed film had strong intermolecular interactions; the molecules in the film were closely arranged; and the film was denser and had better stability and compression resistance.

The zero-pressure molecular area and collapse area of TAN/DPPC mixed films were larger than those of TAN/SA mixed films, but the collapse pressure of TAN/SA mixed films was significantly higher than that of TAN/DPPC mixed films. These results reflect that TAN, as a hydrophobic molecule, exerts different effects on SA and DPPC monolayers.

### 2.3. Phase Transition and Compressibility

For mixed films without obvious phase transition regions, it is difficult to directly determine the state of the LB film from the π-A isotherm. The phase transition point can be determined by calculating the surface compression modulus ($C_{S}^{-1}$); that is, the phase transition process can be inferred from the minimum value in the $C_{S}^{-1}$-π curve. The value of $C_{S}^{-1}$ can be calculated from the slope of the π-A curve by Equation (1) [37,38]:

(1) $$C_{S}^{-1}=-A{\left(\frac{d\pi }{dA}\right)}_{T}$$

where A represents the mean molecular area, and π represents the surface pressure.

**Figure 4:**
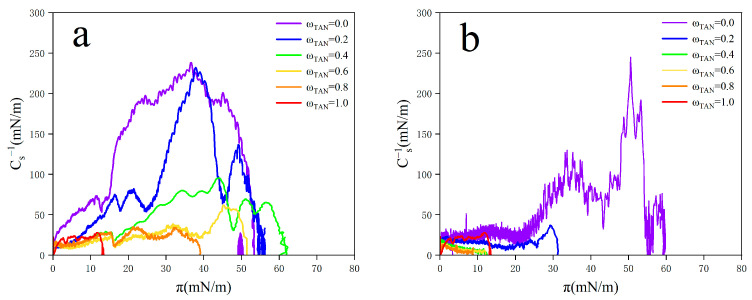
The surface compression modulus of mixed monolayers with different TAN contents: (**a**) TAN/SA, (**b**) TAN/DPPC.

The surface compression modulus curves of mixed TAN/SA and TAN/DPPC films are presented in Figure 4a and Figure 4b, respectively. We can see that the $C_{S}^{-1}$-π curves increased first and then decreased with an increase in surface pressure. The maximum value of $C_{S}^{-1}$ decreased with an increase in TAN content, and there were some extreme values on the curves.

The curve of ω_TAN_ = 0.0 in Figure 4a is the surface compression modulus curve of pure stearic acid. When the surface pressure reached 14 mN/m, the compression modulus of the stearic acid monolayer reached a minimum value of 51 mN/m, and the monolayer began to change from a liquid-expanded film to a liquid-condensed film. In general, the $C_{S}^{-1}$ of the liquid-expanded phase (LE) was 12.5–50 mN/m; the liquid-expanded–liquid-condensed coexistence phase (LE-LC) was 50–100 mN/m; and the liquid-condensed phase (LC) was 100–250 mN/m [39]. There was also a minimum $C_{S}^{-1}$ of 180 mN/m at a surface pressure of 28 mN/m. At this time, the mixed film underwent a phase transition from a liquid-condensed film to a solid film ($C_{S}^{-1}$ > 250 mN/m [39]). Therefore, it can be seen from the surface compression modulus curve that the single-molecular film of pure stearic acid underwent a transition process from low-density film, liquid-expanded film to liquid-condensed film and gradually to solid film with the change in surface pressure. When ω_TAN_ = 0.2, the compression modulus had two minimum values of 51 mN/m and 52 mN/m at surface pressures of 18 mN/m and 25 mN/m, respectively. This was the phase transition of the mixed film from the liquid-expanded film to the liquid-condensed film. The maximum value of $C_{S}^{-1}$ was approaching 250 mN/m, indicating that the phase transition process of this mixed film was similar to that of pure stearic acid. When ω_TAN_ = 0.4, 0.6 and 0.8, the compression modulus of TAN/SA mixed films had a minimum value near the surface pressure of 16 mN/m. When ω_TAN_ = 1, the maximum compression modulus of the monolayer was about 13 mN/m, and it collapsed at a surface pressure of 16 mN/m. As the TAN content increased, the maximum compression modulus of the mixed films also gradually decreased. The maximum $C_{S}^{-1}$ values of ω_TAN_ of 0.4, 0.6 and 0.8 were 100, 62 and 35 mN/m, respectively. Therefore, we can see that, with the increase in TAN content, the phase transition of the mixed film changed from the three phases of low-density film, liquid-expanded film and liquid-condensed film to the two phases of low-density film and liquid-expanded film until the system only formed a low-density film. The phase transition behavior was increasingly influenced by TAN content; TAN reduced the film rigidity and enhanced the fluidity.

The curve of ω_TAN_ = 0.0 in Figure 4b represents the surface compression modulus curve of DPPC. When the surface pressure reached 21 mN/m, the $C_{S}^{-1}$ of DPPC monolayer was 16 mN/m, and it began to change from the liquid-expanded phase to the coexistence of liquid-expanded and liquid-condensed phases. The minimum value of $C_{S}^{-1}$ was about 43 mN/m at the surface pressure of 32 mN/m, the film changed from the coexistence phase to the liquid-expanded phase, and the maximum value of $C_{S}^{-1}$ was 245 mN/m. Therefore, the single-molecular film of DPPC underwent a transition process from low-density film, liquid-expanded film to liquid-condensed film and gradually to solid film with the change in surface pressure. When ω_TAN_ = 0.2, the maximum compression modulus of the mixed film was only 37 mN/m, which was a liquid-expanded film. For TAN/DPPC mixed films with ω_TAN_ = 0.4–0.8, there was no obvious phase transition point in the surface compression modulus curve, and the maximum $C_{S}^{-1}$ was lower than 20 mN/m. The mixed films were also liquid-expanded films.

**Figure 5:**
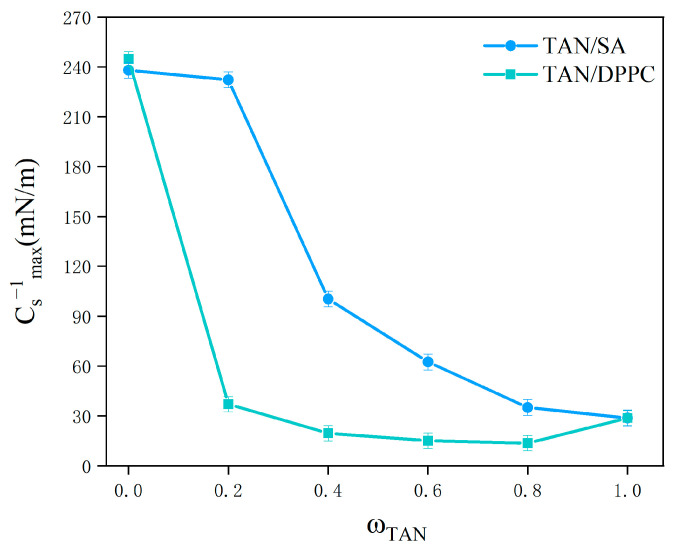
The maximum surface compression modulus of mixed TAN/SA and TAN/DPPC monolayers with different TAN contents.

The surface compression modulus not only reflects the phase transition of the films but also reflects the static elasticity and deformation resistance of the mixed films. A larger compression modulus indicates higher rigidity, stronger deformation resistance, and lower compressibility [40]. Therefore, we summarized the maximum surface compression modulus of TAN/SA and TAN/DPPC mixed films with the change in TAN content, and the results are shown in Figure 5. It can be seen that, in the two mixed system films, the maximum surface compression modulus decreased with an increase in TAN mass fraction, indicating that the incorporation of TAN reduced film rigidity and deformation resistance while increasing the fluidity of both SA and DPPC monolayers. In TAN/SA and TAN/DPPC binary mixed systems, the maximum surface compression modulus of the TAN/SA monolayer film was higher than that of the TAN/DPPC mixed monolayer film at the same mass fraction. In addition, it is worth noting that, in the TAN/DPPC mixed system, the maximum compression modulus of the mixed system was close to or lower than the maximum compression modulus of the pure TAN monolayer, indicating that the mixed films possess high fluidity and good compressibility. These results indicate that the compressive elasticity and phase transition behavior of mixed monolayers are closely related to the interaction between additives and TAN [32].

### 2.4. Shrinkage and Expansion

When analyzing the shrinkage and expansion of the mixed monolayers, we encountered a challenge: the collapse pressure of tangeretin is relatively low, limiting the depth of single-molecule area analysis. The collapse pressure is a key index to measure the stability of the material under the action of surface tension. Its low value means that it is difficult to maintain the structure of the monolayer in the experiment, which brought great difficulties to our single-molecule area analysis. In order to continue to advance the understanding of the surface properties of tangeretin, we turned to the analysis of excess surface pressure.

The shrinkage and expansion of the mixed monolayers can be analyzed by the excess surface pressure (Δπ_ex_), and the calculation formula of Δπ_ex_ is as follows (Equations (2) and (3)) [41,42]:

π_A,ideal_ = x_1_(π_1_)_A_ + x_2_(π_2_)_A_ (2)

Δπ_ex_ = π_ac_ − π_A, ideal_ (3)

**Figure 6:**
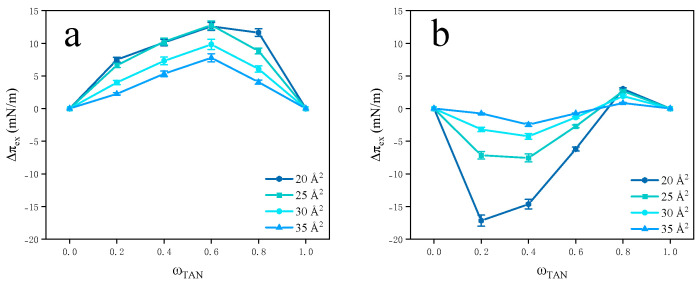
Excess surface pressures of mixed monolayers with different TAN content: (**a**) TAN/SA, (**b**) TAN/DPPC.

In the formula, π_A, ideal_ represents the surface pressure when the two components are ideally mixed. (π_1_)_A_ and (π_2_)_A_ are the surface pressures of each component at a fixed monolayer area. x_1_ and x_2_ are the mole fractions of components 1 and 2 in mixed film (x_1_ + x_2_ = 1). π_ac_ is the actual surface pressure of the mixed film. Δπ_ex_ is the excess surface pressure of the mixed film. The results of the excess surface pressure curve for the mixed film are shown in Figure 6. Among them, the mass fraction has been converted into the corresponding molar fraction during the calculation process.

From Figure 6a, it can be observed that Δπ_ex_ ≠ 0, indicating that the two components, TAN and SA, were non-ideally mixed. At the selected molecular areas, Δπ_ex_ of the TAN/SA mixed films were all positive, suggesting that the intermolecular interactions within the mixed films were predominantly repulsive, and all mixed systems formed expanded films. When ω_TAN_ < 0.6, Δπ_ex_ gradually increased with increasing TAN mass fraction, indicating enhanced repulsive interactions. At ω_TAN_ = 0.6, the maximum Δπ_ex_ was 12.77 mN/m; the repulsive interaction reached its maximum; and the film exhibited the loosest molecular packing. When ω_TAN_ > 0.6, Δπex gradually decreased with further increases in TAN mass fraction, indicating weakened repulsive interactions. For 0.2 < ω_TAN_ < 0.8, at the same TAN mass fraction, Δπ_ex_ of the mixed films decreased as the molecular area increased, suggesting that the intermolecular forces within the mixed films weakened with increasing molecular area.

From Figure 6b, it can be observed that the two components, TAN and DPPC, were also non-ideally mixed. The TAN/DPPC mixed film exhibited a positive Δπ_ex_ at ω_TAN_ = 0.8, indicating that the mixed film was in an expanded state, with intermolecular interactions dominated by repulsive forces. In addition, for all other TAN/DPPC mixed systems, Δπ_ex_ values were negative across the selected molecular areas, suggesting that the intermolecular interactions within the mixed films were primarily attractive, resulting in a condensed film state. The attractive interactions between the two components were likely governed by hydrogen bonding between the amino group of DPPC and the p-π conjugated bonds of TAN. At the same TAN mass fraction, the absolute value of the excess surface pressure decreased as the molecular area increased, indicating that the intermolecular forces weakened with the increase in the molecular area. At the different molecular areas, the minimum excess surface pressure (−17.16 mN/m) was observed at ω_TAN_ = 0.2, indicating that the attraction in the mixed film was the strongest, and the film was the densest. When 0.4 ≤ ω_TAN_ ≤ 0.8, the absolute value of the excess surface pressure gradually decreased with increasing TAN mass fraction, indicating weakened attractive interactions and even the emergence of repulsive forces. This suggests that a small amount of TAN enhances the tightness of the molecules in the DPPC film, making the mixed film more condensed, whereas excessive TAN leads to film loosening.

Comparing the Δπ_ex_ of the two mixed systems, the TAN/DPPC mixed films exhibited attractive intermolecular interactions and demonstrated better miscibility at low TAN concentrations. In contrast, the TAN/SA mixed films showed repulsive intermolecular interactions, resulting in poorer mixing behavior.

### 2.5. Thermodynamic Stability

The thermodynamic stability and intermolecular interactions (repulsion or attraction) of mixed monolayer membranes can be studied by excess Helmholtz free energy (${\Delta A}_{m}^{ex}$). The calculation formula is shown in Equation (4) [42,43,44]:

(4) $${\Delta A}_{m}^{ex}=N_{A}\int_{A_{0}}^{A}(\pi_{12}-x_{1}\pi_{1}-x_{2}\pi_{2})dA$$

Here, A_0_ is the single-molecule area corresponding to the increase in π starting from π = 0; A is the single-molecule area for calculating ${\Delta A}_{m}^{ex}$; π_12_ is the surface pressure of the mixed single-molecule film obtained during the experimental film pressing process; π_1_ = (π_1_)A and π_2_ = (π_2_)A are the surface pressures of a single component at a certain single-molecule area; x_1_ and x_2_ represent the molar fractions of component 1 and component 2 (x_1_ + x_2_ = 1); N_A_ is the Avogadro constant.

**Figure 7:**
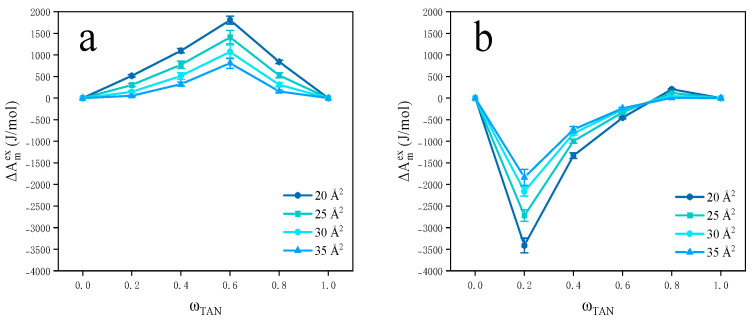
The excess Helmholtz free energy values of mixed monolayers with different TAN contents: (**a**) TAN/SA, (**b**) TAN/DPPC.

Figure 7 is the result of ${\Delta A}_{m}^{ex}$ of TAN/SA and TAN/DPPC mixed films with different single-molecule areas (20, 25, 30, 35 Å^2^). The mass fraction was converted into the corresponding molar fraction during the calculation process.

From Figure 7a, it can be observed that all the ${\Delta A}_{m}^{ex}$ were positive at all molecular areas, indicating that the intermolecular interactions between TAN and SA were repulsive. This finding is consistent with the analysis results of the excess surface pressure. This phenomenon might be attributed to the incorporation of TAN into the SA monolayer, which weakened the intermolecular interactions among SA molecules and reduced the thermodynamic stability of the system. As the molecular area decreased, ${\Delta A}_{m}^{ex}$ of the mixed systems at the same TAN mass fraction gradually increased, suggesting a progressive reduction in the stability of the mixed system. When ω_TAN_ = 0.6, ${\Delta A}_{m}^{ex}$ reached its maximum, and the value was 1805.66 J/mol, indicating that this system exhibited the poorest thermodynamic stability [44].

From Figure 7b, we observe that ${\Delta A}_{m}^{ex}$ exhibited negative values within the range of 0.2 ≤ ω_TAN_ ≤ 0.6, and its absolute value increased with a decrease in molecular area. This indicates that the reduction in molecular area enhanced the attractive interactions between DPPC and TAN molecules, leading to increased spontaneity and improved stability of the mixed system. When ω_TAN_ = 0.2, all mixed systems reached the minimum value of ${\Delta A}_{m}^{ex}$ at different molecular areas, and the minimum ${\Delta A}_{m}^{ex}$ was −3413.62 J/mol. This suggests that the p-π conjugated bond between the two components was strongest at ω_TAN_ = 0.2, resulting in the highest thermodynamic stability of the mixed monolayer. When 0.2 < ω_TAN_ ≤ 0.6, the absolute value of ${\Delta A}_{m}^{ex}$ gradually decreased with increasing TAN mass fraction, indicating a reduction in system stability. At ω_TAN_ =0.8, ${\Delta A}_{m}^{ex}$ became positive across all selected molecular areas and decreased as the molecular area increased. This demonstrates that the mixed monolayer exhibited lower thermodynamic stability with repulsive intermolecular interactions between TAN and DPPC, which is consistent with the analysis of excess surface pressure.

Based on the analysis of shrinkage/expansion and thermodynamic stability in TAN/SA and TAN/DPPC mixed systems, consistent results were obtained that both binary mixtures exhibited partial miscibility in the mixed films. The TAN/SA mixed films demonstrated intermolecular repulsion and phase separation, which align with the results reported by Ikbal et al. [45] and He et al. [46], confirming the existence of repulsive interactions between molecules. In contrast, the TAN/DPPC binary monolayer showed attractive interactions between components, a finding consistent with the thermodynamic stability curves observed by Marta et al. [47], indicating more-favorable intermolecular interactions.

The above analysis confirms that the TAN/DPPC mixed system at ω_TAN_ = 0.2 exhibits the strongest attractive intermolecular interactions and optimal thermodynamic stability. It is noteworthy that DPPC has been extensively employed as a biocompatible anchoring lipid in various drug delivery platforms, including mixed micelles and inhalable lipid nanoparticles, to solubilize poorly water-soluble drugs and improve their bioavailability [48,49]. The favorable mixing behavior between TAN and DPPC identified in this work suggests that DPPC-based nanocarriers could be rationally designed to encapsulate tangeretin for therapeutic applications. Furthermore, given the pronounced antioxidant and anti-inflammatory activities of tangeretin, the interfacial assembly approach studied here may also be extended to the immobilization of proteins or enzymes in Langmuir–Blodgett films for biosensing purposes, as recently demonstrated with chitinase and other enzymes [50,51]. These perspectives significantly broaden the scope of tangeretin-loaded phospholipid monolayers beyond drug delivery and highlight their potential in multifunctional interfacial platforms.

## 3. Materials and Methods

### 3.1. Materials

Tangeretin (TAN) was purchased from ChemFaces (Wuhan, China). Stearic acid (SA) and chloroform were purchased from Beijing Chemical Factory (Beijing, China). Dipalmitoyl phosphatidyl choline (DPPC) was purchased from Med Chem Express LLC (Monmouth Junction, NJ, USA). Methanol was purchased from Fuchen (Tianjin) Chemical Reagent Co., Ltd. (Tianjin, China). All the above materials were analytically pure. For all experiments, ultrapure water (resistivity = 18 MΩ cm) was used as the subphase and used to clean the tank.

### 3.2. Methods

Stock solutions of TAN, SA, and DPPC were individually prepared in methanol/chloroform (3:7, *V*/*V*) at 0.5 mg/mL. These were mixed in appropriate ratios to obtain spreading solutions with TAN mass fractions ranging from 0 to 100 wt%, while maintaining a constant total film-forming material concentration of 0.5 mg/mL. For each experiment, precisely 10 μL of spreading solution was deposited dropwise onto the ultrapure water subphase at room temperature. After 15 min for solvent evaporation and film equilibration, the monolayer was compressed at a constant barrier speed of 10 mm/min using a Langmuir–Blodgett film analyzer (Micro Trough, Kibron, Helsinki, Finland). Surface pressure was continuously recorded by the Wilhelmy plate technique, and π-A isotherms were constructed. In all measurements, the total mass of film-forming material was kept constant (5 μg), while the total number of molecules varied with composition; this constant-mass design was adopted to evaluate mixed systems at a fixed material loading. Each isotherm was measured at least three times.

## 4. Conclusions

The study systematically investigated the isotherm analysis, characteristic parameters, surface compression modulus, excess surface pressure and excess Helmholtz free energy of the mixed monolayers of TAN/SA and TAN/DPPC. The results demonstrated that TAN significantly influenced the monolayer properties of SA and DPPC and exhibited different mixed film properties due to the differences in interaction between TAN and SA or DPPC molecules. The zero-pressure molecular area of TAN/DPPC mixed films showed a trend of first increasing and then decreasing with an increase in TAN mass fraction. When ω_TAN_ = 0.4, the zero-pressure molecular area of the mixed films reached the maximum value of 61 Å^2^. In the TAN/DPPC mixed system, the maximum compression modulus was comparable with or slightly lower than that of pure TAN monolayers. The strong attractive interactions between TAN and DPPC molecules resulted in condensed, compressed mixed films with excellent deformation resistance and good compressibility. In contrast, the TAN/SA mixed films at equivalent mass concentrations exhibited repulsive intermolecular interactions, forming loose monolayers with higher rigidity and poor compressibility. The strongest attractive interactions between TAN and DPPC molecules were observed at ω_TAN_ = 0.2, at which the mixed monolayer exhibited the strongest deformation resistance and optimal stability, and the minimum excess surface pressure (Δπ_ex_) and excess Helmholtz free energy (${\Delta A}_{m}^{ex}$) of TAN/DPPC system were −17.16 mN/m and −3413.62 J/mol, respectively. The maximum Δπ_ex_ and ${\Delta A}_{m}^{ex}$ of TAN/SA system were 12.77 mN/m and 1805.66 J/mol, respectively. This research provides important theoretical support and experimental evidence for understanding the mixed monolayer systems of TAN with SA or DPPC molecules. The findings establish a theoretical foundation for future studies on more-complex and complete film systems, potentially facilitating broader pharmacological applications of TAN.

## Data Availability

The data presented in this study are available on request from the corresponding author.
